# Changes in Predicted Muscle Coordination with Subject-Specific Muscle Parameters for Individuals after Stroke

**DOI:** 10.1155/2014/321747

**Published:** 2014-06-25

**Authors:** Brian A. Knarr, Darcy S. Reisman, Stuart A. Binder-Macleod, Jill S. Higginson

**Affiliations:** ^1^Delaware Rehabilitation Institute, STAR Health Sciences Complex, University of Delaware, 540 S. College Avenue, Newark, DE 19716, USA; ^2^Department of Physical Therapy, STAR Health Sciences Complex, University of Delaware, 540 S. College Avenue, Newark, DE 19716, USA; ^3^Department of Mechanical Engineering, STAR Health Sciences Complex, University of Delaware, 540 S. College Avenue, Newark, DE 19716, USA

## Abstract

Muscle weakness is commonly seen in individuals after stroke, characterized by lower forces during a maximal volitional contraction. Accurate quantification of muscle weakness is paramount when evaluating individual performance and response to after stroke rehabilitation. The objective of this study was to examine the effect of subject-specific muscle force and activation deficits on predicted muscle coordination when using musculoskeletal models for individuals after stroke. Maximum force generating ability and central activation ratio of the paretic plantar flexors, dorsiflexors, and quadriceps muscle groups were obtained using burst superimposition for four individuals after stroke with a range of walking speeds. Two models were created per subject: one with generic and one with subject-specific activation and maximum isometric force parameters. The inclusion of subject-specific muscle data resulted in changes in the model-predicted muscle forces and activations which agree with previously reported compensation patterns and match more closely the timing of electromyography for the plantar flexor and hamstring muscles. This was the first study to create musculoskeletal simulations of individuals after stroke with subject-specific muscle force and activation data. The results of this study suggest that subject-specific muscle force and activation data enhance the ability of musculoskeletal simulations to accurately predict muscle coordination in individuals after stroke.

## 1. Introduction

Musculoskeletal simulations have the potential to provide insight into muscle coordination and function for individuals with gait deficits. Previous musculoskeletal simulations have shown how muscle coordination can be altered based on changes in muscle properties [1–4]. A current limitation of musculoskeletal simulations, however, is that the appropriate muscle properties to use for a specific individual are unknown. For a particular subject or population (e.g., stroke), muscle parameters may differ greatly from default model values, and it has been suggested that selection of muscle parameters can have a relevant impact on simulation results [5–7].

Muscle weakness, characterized by lower forces during a maximal volitional contraction, is a major limiting factor affecting performance of poststroke gait [8]. The two main causes of poststroke muscle weakness are disuse atrophy [9] and impaired muscle activation by the central nervous system [10]. Studies have shown a reduction in skeletal muscle mass and an increase in intramuscular fat in the paretic limb of stroke survivors [9, 11]. Additionally, electromyography (EMG) has been used to demonstrate activation impairment in stroke survivors, with measured EMG amplitude lower on the paretic side muscles compared to the nonparetic side [12]. More recently, studies have used the burst superimposition technique, which applies electrical stimulation superimposed over a volitional contraction, to measure subject-specific maximum force generation ability and volitional activation ratio of muscles for healthy and poststroke populations [13–16].

A study by Xiao and Higginson (2010) explored the sensitivity of a musculoskeletal model to changes in muscle parameters, showing that predicted muscle forces are sensitive to values of tendon slack length, optimal fiber length, and differences greater than 10% in maximum isometric force [3]. Strength deficits seen after stroke are often in excess of 10%, with previous studies reporting paretic side voluntary moment 80% less than nonparetic force for some individuals [15–18]. Since variation of muscle properties influences muscle force and coordination, it is possible that the inclusion of relevant muscle parameters in musculoskeletal models will lead to more accurate and meaningful results in persons after stroke.

It has been shown in previous work that model predictions of muscle coordination are altered when muscle weakness is simulated [1, 4]; however, these studies only involved randomly imposed weakness to healthy simulations. To date, no studies have built subject-specific musculoskeletal models which include experimentally measured values for muscle weakness from a clinical population such as individuals after stroke. Therefore, the objective of this study was to examine the effect of subject-specific muscle force and activation deficits on muscle coordination when using musculoskeletal models for individuals after stroke. Three-dimensional subject-specific musculoskeletal models were built using experimental gait data from subjects after stroke. Two simulations were created per subject, one using generic and one using subject-specific isometric force and maximum volitional activation model parameters based on experimentally measured data. We hypothesized that subject-specific activation and muscle force data would result in altered predicted muscular control patterns that are consistent with muscle compensation strategies that have been reported in both modeling and clinical studies. Additionally, we hypothesized that the timing of the subject-specific activations predicted by the musculoskeletal model would agree better with the timing of experimentally recorded electromyography measured during gait when subject-specific model parameters were used.

## 2. Methods

Four individuals after stroke (65 ± 8 yrs, 9 ± 4 months after stroke) were recruited to participate in this study. Subjects were included in this study if they met the following criteria: 6 months after a stroke involving cerebral cortical regions, being able to walk for 5 minutes at self-selected speed without a brace or assistive device, passive paretic ankle dorsiflexion range of motion to reach at least 5° of plantar flexion with the knee flexed, and presence of deficits in walking function. Subjects were excluded from the study using the following criteria: severe aphasia, substantial cognitive deficits, cerebellar involvement, or preexisting conditions affecting walking function [19]. All subjects signed informed consent forms approved by the Institutional Review Board at the University of Delaware.

Kinematic (60 Hz) and kinetic (1080 Hz) gait data were collected using an 8-camera motion capture system (Motion Analysis Corp., Santa Rosa, CA, USA) as subjects walked without a brace or assistive device at their self-selected walking speed on an instrumented split-belt treadmill (Bertec, Columbus, OH). Kinematics and kinetic data were filtered at 6 Hz. Self-selected walking speed was determined using the average of three trials of the 6-meter walk test during over ground walking. Subjects wore an overhead support harness with no body weight support and were instructed to use handrails only as necessary.

Electromyography (EMG) data was recorded during the walking trials. Electrodes were placed on 9 paretic side muscles by palpation of the muscle bellies (Table 1) and were tested manually through resisted motion. EMG data were collected at 1080 Hz and filtered in postprocessing with an 8th order zero-phase shift low-pass Butterworth filter with a cut-off frequency of 20 Hz high-pass filter. The EMG data were rectified and then filtered with an 8th order zero-phase shift low-pass Butterworth filter with a cut-off frequency of 5 Hz. An eight-order filter was selected as it gave the cleanest signal for activation onset and offset detection. The EMG data were normalized to the peak EMG signal from the walking trial and a representative gait cycle was selected and interpolated to 100 points.

### 2.1. Muscle Parameter Testing

Subjects performed the burst superimposition test to assess the maximum force generating ability (MFGA) and central activation ratio (CAR) of the paretic side plantar flexors, dorsiflexors, and quadriceps muscle groups. Previously developed adjustment equations were applied to the result of the burst superimposition test to improve the accuracy and reliability of the test at submaximal volitional effort [13–15]. For testing of the plantar flexor muscle group, subjects lay supine on a KIN-COM III dynamometer (Chattecx Corp, Chattanooga, Tennessee) with their knee in extension and ankle at neutral. Velcro straps were used to hold the foot and shank in position and restraints were placed on the shoulders of the subject to ensure that all forces were directed into the transducer and not lost to body displacement. During the burst superimposition test, an initial maximal single pulse (600 *μ*s, 135 V) was delivered to the resting muscle. After this pulse, subjects had five seconds to reach their maximum volitional effort. Five seconds after the first pulse, a maximal electrical stimulation burst (600 *μ*s pulse duration, 100 ms train duration, 135 V, 100 Hz train) was delivered while subjects produced their maximum volitional force. Predicted maximum force generating ability (MFGA), or the force produced with full muscle activation, using the burst superimposition test (MFGA_burst_) was calculated using the following equation:

(1) $$\begin{array}{c} {\text{MFGA}}_{\text{burst}}=F_{\text{vol}}+F_{\text{stim}}, \end{array}$$

where *F*_vol_ was the volitional force produced by the subject and *F*_stim_ was the additional force produced by the stimulation. A cubic adjustment was then applied to the MFGA prediction to account for low levels of volitional activation [13] when testing the plantar flexor muscles. The level of volitional muscle activation, called the central activation ratio (CAR), is calculated as the ratio of volitional force (*F*_vol_) to the maximum force generating ability of a muscle or muscle group (MFGA) (2)

(2) $$\begin{array}{c} \text{CAR}=\frac{F_{\text{vol}}}{{\text{MFGA}}_{\text{burst}}}, \end{array}$$

where MFGA_burst_ is the MFGA predicted through the burst superimposition method used in this study.

Muscle testing was repeated for the dorsiflexors while laying supine with the ankle at 15° of plantar flexion. For testing of the quadriceps muscle group subjects were seated upright with their hip at 90 degrees and their knee at 60 degrees. A previously developed adjustment was applied to the maximal force prediction for the quadriceps [14, 20] to account for low levels of volitional activation during the test.

### 2.2. Musculoskeletal Simulations

Two musculoskeletal models were created in OpenSim [21] per subject: one with generic and one with subject-specific activation and maximum isometric force parameters. The musculoskeletal model included 54 actuators, with three degrees of freedom at the pelvis and hip joints and one degree of freedom at the knee, ankle, and toe joints. The model was scaled to the subject's size and mass. Model joint kinematics were determined using inverse kinematics to determine the model position which best matches the experimental marker data. Kinematics and kinetic data were filtered at 6 Hz. Additionally, the residual reduction algorithm was run to minimize the residual forces that account for dynamics inconsistencies between kinematic and kinetic data. Residual and reserve actuators were added to the model. These actuators account for forces the model could not resolve with muscle actuators alone. Simulations were created from heel strike to heel strike of the paretic limb and all data were reported for the paretic limb. Using the subject-specific force and activation data obtained through burst superimposition testing, the maximum isometric force and maximum activation parameters of the model were set for the quadriceps (rectus femoris and vastus intermedius), plantar flexors (soleus, medial gastrocnemius, and tibialis posterior), and dorsiflexors (tibialis anterior) of the paretic limb. The maximum activation of each muscle in a muscle group was set uniformly. For subjects that could not volitionally dorsiflex, the burst superimposition test was not performed, and a maximum activation of 0.02 was used in the model, where model activation ranges from 0 to 1. A value of 0.02 was used because it is the minimum activation the model will allow. The total isometric force produced by the group was distributed proportionally to each of the muscles in the model according to the ratios of the maximum isometric muscle force within the muscle group in the default model.

Using the computed muscle control (CMC) [22] algorithm in OpenSim [21], the muscle forces and activations required to reproduce the experimental kinetics and kinematics were calculated. To quantify the difference in muscle activation between the two models, the average level of activation was calculated over the full gait cycle and during the double support phase, defined as the period of double support on the paretic limb during preswing. The double support phase was chosen as it has been a focus of recent studies for gait rehabilitation [23] and musculoskeletal modeling of poststroke gait [24, 25].

Onset and offset of the musculoskeletal model muscle activations were compared to the onset and offset of EMG data collected during the walking trial. Visual inspection was used for determining onsets and offsets of model activation and EMG data due to poor performance with the highly variable poststroke EMG data by the traditional threshold detection method of multiple standard deviations above a baseline value [26]. Visual inspection was repeated twice to ensure that the on-/off-times were consistent. For each point, the graph was zoomed to pick the first point rising/last point falling during the beginning and end of onset period. Timing agreement with EMG, defined as the portion of the gait cycle during which the model-predicted activation was either on or off at the same time as the EMG, was calculated for the activation timing of the generic and subject-specific models. Timing agreement varied from 0 to 1, with a value of 1 indicating complete agreement between the EMG and model activation.

### 2.3. Statistical Analysis

Due to the number of subjects in this study, traditional statistical methods were not applicable. Instead, we assert that a change of greater than ±0.05 in activation or greater than ±50 N in force per muscle for at least three of the four subjects should be considered a meaningful change as a result of the inclusion of subject-specific parameters. A change of 0.05 in activation was based on the minimum level of significant change in model activation that has been reported in a muscle after a targeted rehabilitation protocol for individuals after stroke (0.045) [25]. A 50 N change was also imposed to ensure that the muscle force was changing in conjunction with an increase in activation.

## 3. Results

A total of eight simulations were generated, with simulations using generic and subject-specific maximum isometric force and maximum activation parameters created for each of the four subjects (Table 2).

### 3.1. Muscle Activation

**Figure 1:**
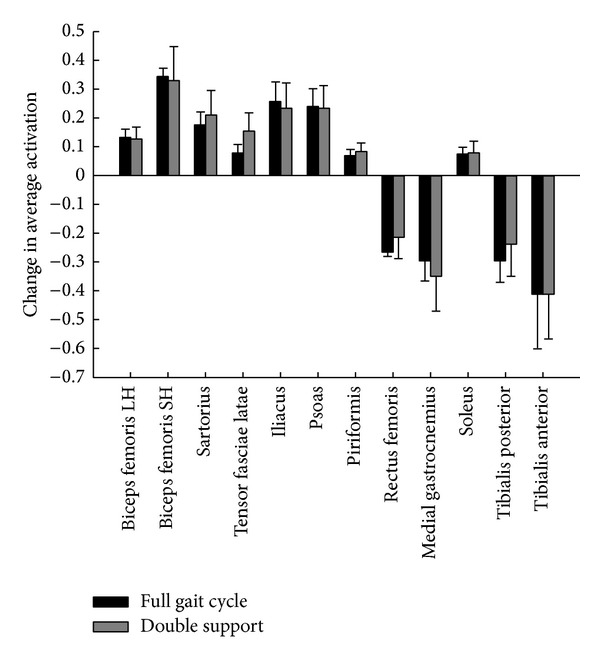
Average change in activation across 4 subjects with the addition of subject-specific maximum isometric force and maximum activation parameters for the 12 paretic side muscles with changes greater than 5% for at least 3 of 4 subjects. Subject-specific model activation limited to 0.02 for subjects unable to volitionally dorsiflex during isometric testing.

Maximum activation, as assessed through CAR, was lower in the subject-specific models than the generic model for all three muscle groups tested (quadriceps 0.41  ±  0.14 maximum activation, plantar flexors 0.32 ± 0.20 maximum activations) (Table 3). Twelve muscles showed changes greater than 0.05 in average activation for at least three of the four subjects over either the full gait cycle or double support phase (Figure 1) when subject-specific muscle parameters were used. The hip flexors (iliacus, psoas) and knee flexors (biceps femoris short head, sartorius) showed the greatest increases in activation over both the full gait cycle and double support phase (0.24, 0.23, 0.33, and 0.21 during double support, resp.). The knee extensors (rectus femoris), plantar flexors (medial gastrocnemius, tibialis posterior), and dorsiflexors (tibialis anterior) showed average decreases in activation over both the full gait cycle and double support (−0.21, −0.35, −0.24, and −0.41 over double support, resp.). Soleus activation increased in the subject-specific models, compensating for decreased medial gastrocnemius and tibialis posterior activation.

### 3.2. Muscle Force

**Figure 2:**
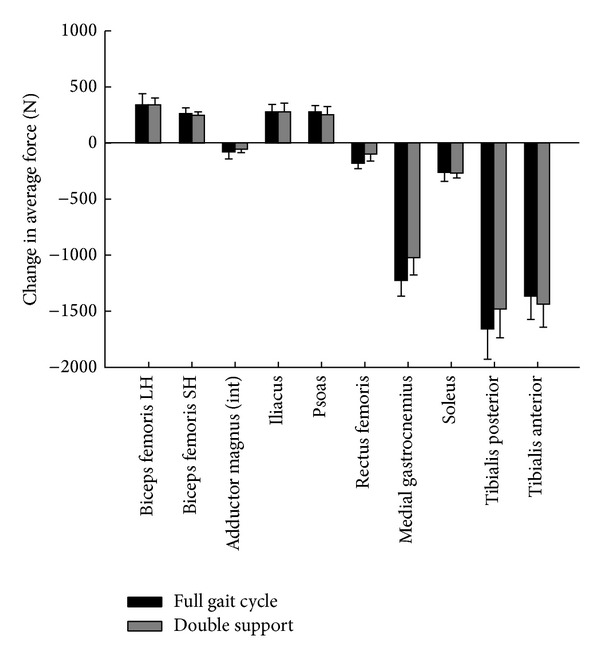
Average change in force across 4 subjects with the addition of subject-specific maximum isometric force and maximum activation parameters for the 10 paretic side muscles with changes greater than 50 N for at least 3 of 4 subjects. Int = intermediate.

Maximum isometric force was 1.87 ± 0.37 times greater in the quadriceps and 0.24 ± 0.08 times lower in the plantar flexor muscle group for the subject-specific model (Table 3). Ten muscles showed changes greater than 50 N in average force for at least three of the four subjects over either the full gait cycle or double support phase (Figure 2) when subject-specific muscle parameters were used. The hip flexors (iliacus, psoas) and knee flexors (biceps femoris long and short head) showed the largest increase in force (272, 271, 334, and 356 N in double support, resp.), while the plantar flexors (medial gastrocnemius, tibialis posterior, and soleus) and dorsiflexors (tibialis anterior) showed the largest decrease in force over both the full gait cycle and double support (−1228, −1659, −265, and −1367 N in double support, resp.).

### 3.3. Muscle Activation Timing

**Figure 3:**
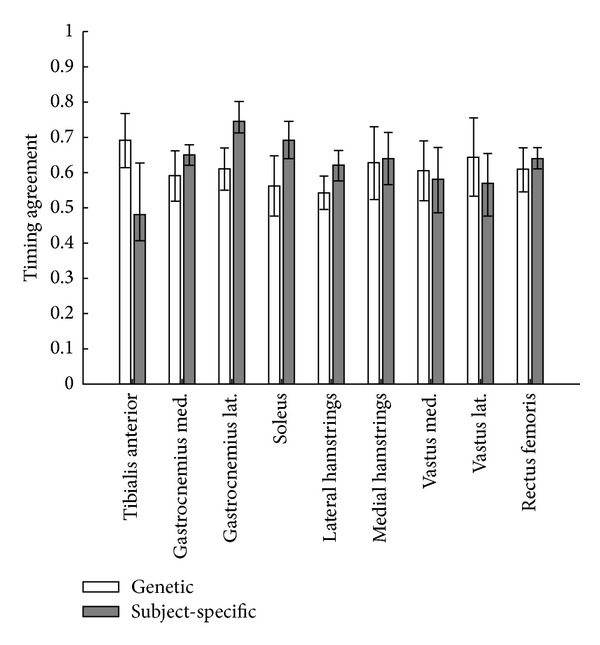
Timing agreement between EMG and model activation when using generic or subject-specific muscle parameters for the nine muscles with EMG collected. Greater value indicates better timing agreement. Med = medial, Lat = lateral. Subject-specific model activation limited to 0.02 for subjects unable to volitionally dorsiflex during isometric testing.

EMG and model activation timing agreement generally ranged from 0.5 to 0.8 for the muscles collected. Agreement of model-predicted muscle activation timing with EMG increased for the plantar flexors (0.06, 0.15, and 0.13, for the medial and lateral gastrocnemius and soleus, resp.) and biceps femoris (0.08) when subject-specific parameters were used (Figure 3). Agreement decreased for the tibialis anterior (−0.21) and vastus lateralis (−0.07) muscles. Decreased agreement of the tibialis anterior is likely due to subjects' inability to volitionally dorsiflex during isometric testing, limiting subject-specific model activation to 0.02. EMG of the tibialis anterior muscles for one subject and lateral hamstrings and vastus lateralis for a second subject were not used due to poor signal quality.

## 4. Discussion

Including subject-specific parameters for maximal force generating ability and activation when using musculoskeletal models to assess muscle coordination in individuals after stroke had important implications for model predicted activation, force, and coordination. Nine muscles showed a change in both average activation and force over double support or the full gait cycle. Increases were seen in the activation and force of the hip flexor and knee flexor muscle groups while decreases were seen in the activation and force of the knee extensor, plantar flexor, and dorsiflexor muscle groups when subject-specific muscle parameters were used.

Our experimental results indicate that the subjects in our study had considerably less plantar flexor and dorsiflexor maximum force generating ability and volitional activation than represented by the generic OpenSim muscle properties (Table 3). Plantar flexor weakness in this study is consistent with previous studies, which show volitional activation impairment [15–18] and lower maximum force generating ability [15, 16] in individuals after stroke. Three subjects in our study exhibited no volitional activation of the dorsiflexors during burst superimposition testing, which supports findings of inadequate dorsiflexor activation as common limitation to poststroke gait [12]. Although quadriceps was less impaired than the dorsiflexors and plantar flexors, its CAR was still less than CAR reported for older adults [27] and the maximum force generating ability of the quadriceps was within the range of previously reported data for older adults [20].

Previously, model results were shown to be sensitive to differences greater than 10% in maximum isometric force [3]. With substantial weakness known to occur in individuals after stroke [16–18] and greater than 80% for some muscles and subjects in this study, we would expect substantial differences between the generic and subject-specific models. Therefore, the changes seen in the model predictions with the inclusion of subject-specific parameters in this study were not unexpected.

Recent studies have shown the ability of the hip flexors and knee flexors to compensate for plantar flexor deficiencies [4]. Additionally, greater hip flexor activity has been cited as a compensation seen in individuals following stroke to overcome plantar flexor weakness [28]. Interestingly, the changes seen in model predictions with the addition of subject-specific parameters were consistent with predictions of muscle compensation strategies by previous model-based studies with muscle deficits [1, 4]. Increases in knee flexor activity were also observed in this study and have been reported previously in response to plantar flexor weakness [1]. Increased knee flexor activity is likely a compensation for reduced knee flexion contribution both from the gastrocnemius directly and from induced knee flexion acceleration by the plantar flexors as a group [29]. It is likely that the decrease in rectus femoris activation and force seen in our subject-specific models is the result of multiple factors. First, there is a decreased need for contribution to hip flexion by the rectus femoris because of increased activity of the ipsilateral hip flexors. Second, there is a reduced need for contribution to knee extension by the rectus femoris due to reduced opposing knee flexion generated by the plantar flexors. Overall, the vastus intermedius activation was very low using both models (0.05–0.1), with the biarticular rectus femoris being much more active. As a result, no appreciable change was seen on average in the activation and force between generic and subject-specific models.

The activation timing predicted by the model agreed more closely with EMG for the plantar flexors, biceps femoris, and rectus femoris when the subject-specific muscle parameters were used. Increased agreement in the biceps femoris is noteworthy, as subject-specific parameters were not used for the hamstrings in the model. This shows that the inclusion of subject-specific parameters in some muscles can increase the validity of model predictions for muscles that do not have subject-specific parameters. In contrast, agreement of activation timing with EMG for the tibialis anterior and vastus medialis and lateralis decreased with subject-specific parameters. It is possible that the poor EMG timing agreement for the vastii is due to representing the vastii muscle group together as the vastus intermedius in the model. Future models which individually model vastii muscles may show improved agreement with measured EMG timing. Poor tibialis anterior agreement likely occurred because the tibialis anterior was constrained to “off” (0.02 max activation) for 3 of the 4 subject-specific models. EMG signals could still be measured for the tibialis anterior during gait, however, despite no volitional activation during the isometric testing, causing the discrepancy between EMG and model activation. This suggests that future studies should consider a dynamic test for assessing dorsiflexor muscle properties in a poststroke population, as static isometric testing may not capture a poststroke individual's capacity to volitionally activate the dorsiflexors during gait.

An interesting finding which highlights the passive properties of the muscle-tendon unit was seen at the ankle. In our subjects, there was extreme weakness in the plantar flexors and dorsiflexors, and during the paretic push-off phase of gait the model was actually unable to fully generate the joint torques required using muscle forces alone. As a result of this, a model “reserve” actuator was needed for the paretic side ankle joint to generate the rest of the joint torque required, and for some simulations this reserve actuator torque peaked between 30 and 50 Nm. The ankle reserve actuator was needed only for the subject-specific models, which incorporated the weakness measured for our subjects experimentally. No other reserve actuators contributed meaningful torque in any of simulations. This is interesting particularly because we also observed large amounts of baseline or resting plantar flexor torque when these subjects were undergoing burst superimposition testing. This suggests that these subjects are generating a much larger amount of passive force (as was evident during isometric testing), which would likely play a significant role during walking, and this may have been captured by the model's reserve actuators. Future studies should consider quantifying changes in passive muscle and tendon force and altering modeled muscle properties to more accurately simulate motion of individuals after stroke.

Subject-specific information in this study was limited to the maximum force generating ability and maximum activation of the paretic quadriceps, plantar flexors, and dorsiflexors. Additional muscles and muscle parameters, such as tendon slack length and optimal fiber length, have been shown to influence model results [3] and could be considered in future studies, as they may be influenced by muscle spasticity commonly seen after stroke. The cost function used in our simulations minimizes the sum of the squares of the muscle excitations, which is not necessarily appropriate for poststroke gait and may influence the model results. EMG was not used to constrain the activations predicted by the model so that we could explore whether model activations predicted with the addition of subject-specific parameters would match EMG patterns more closely than the generic model. Muscle performance was tested in an isometric condition, which may not precisely represent the activation capacity of an individual after stroke during a dynamic task such as gait. A limited sample size of four subjects was used for this study; however, the subjects included in this study represented a wide range of function, with self-selected walking speeds ranging from 0.18 to 1.04 m/s. The fact that similar results were found across this range of walking function indicates that including subject-specific parameters is important regardless of the magnitude of functional impairment.

This is the first study to create musculoskeletal simulations of individuals after stroke with subject-specific muscle force and activation data. The inclusion of subject-specific muscle data resulted in changes in the model-predicted force and activation data which agree with previously reported compensation patterns [1, 4, 28]. Additionally, the timing of muscle activation predicted by the model agreed more closely with the timing of EMG for the plantar flexor and hamstring muscles when subject-specific parameters were used. The results of this study suggest that subject-specific isometric force and activation data may affect the accuracy of model predictions and should be used when building musculoskeletal models of individuals after stroke.

## Figures and Tables

**Table 1:** Muscles used for comparison of activation timing between EMG and model predictions.

EMG muscle	Model muscle
Tibialis anterior	Tibialis anterior
Medial gastrocnemius	Medial gastrocnemius
Lateral gastrocnemius	Medial gastrocnemius
Soleus	Soleus
Lateral hamstrings	Biceps femoris long head
Medial hamstrings	Biceps femoris long head
Vastus medialis	Vastus intermedius
Vastus lateralis	Vastus intermedius
Rectus femoris	Rectus femoris

**Table 2:** Subject demographics.

Subject	Sex	Age (yrs)	Weight	Affected side	Time since stroke	Self-selected walking speed (m/s)
287	M	63	91.86	L	7 months	0.18
293	M	54	98.67	L	6 months	1.04
313	M	74	90.49	R	14 months	0.32
314	M	67	81.46	L	9 months	0.65

**Table 3:** Scaling factors used for maximum isometric force and activation for the subject-specific models.

Subject	287		293		313		314	
	Force	Activation	Force	Activation	Force	Activation	Force	Activation
Knee extensors	2.27	0.32	2.09	0.61	1.49	0.39	1.63	0.32
Plantar flexors	0.15	0.16	0.33	0.61	0.18	0.26	0.28	0.23
Dorsiflexors	1.00	0.02	0.05	1.00	1.00	0.02	1.00	0.02
